# Promises and caveats of *in silico* biomarker discovery

**DOI:** 10.1038/sj.bjc.6604495

**Published:** 2008-07-29

**Authors:** L Pusztai, B Leyland-Jones

**Affiliations:** 1 Department of Breast Medical Oncology, The University of Texas, M.D. Anderson Cancer Center, Unit 1354, 1515 Holcombe Boulevard, Houston, TX 77030-1439, USA; 2 Emory University School of Medicine, Atlanta GA, 30322

Several biomarkers have been reported to be associated with survival in breast cancer over the past decades. Unfortunately, it is often difficult to distinguish between the prognostic and predictive values of these proposed markers and independent validations are frequently lacking. Patients with early-stage breast cancer receive various combinations of treatments including surgery, post-operative radiation therapy, adjuvant endocrine treatment and chemotherapy. Each of these can have an impact on survival, and most biomarker studies included heterogeneously treated patient cohorts. The classical methodology of assessing the true prognostic value of a marker was restricted to patients who underwent local regional therapy alone and received no systemic adjuvant treatment.

However, the clinical utility of such prognostic markers in the absence of systemic therapy is limited in this era. Endocrine sensitivity in newly diagnosed stage I–III breast cancer may be best evaluated in patients who received adjuvant endocrine treatment but no chemotherapy after surgery and the chemotherapy response predictive value of a marker may be best examined in the preoperative treatment setting where tumour response can be measured directly. Undoubtedly, the best samples to assess interaction between markers and treatment outcome are from randomized clinical trials that compare different treatment modalities. Unfortunately, many such trials from the past with good outcome data lack tumour banks: it is difficult to obtain specimens from large cohorts of homogeneously treated patients and specimens from randomized clinical trials are an even rarer research commodity. This motivates systematic efforts to collect biological specimens in cancer clinical trials today.

Traditional markers also require separate assays to be performed for each marker on the limited tissue resources. Therefore, it is not surprising that few prognostic or predictive markers have completed the necessary validation steps on the right type of clinical specimens to convince physicians about their clinical value (Simon, 2005). In this issue of the journal, Epping *et al* (2008) report on that PRAME mRNA expression is prognostic in early-stage breast cancer. They observed that this gene had higher expression in tumours that relapsed within 5 years in the absence of systemic adjuvant therapy. The bimodal distribution of PRAME expression offered an opportunity to define a natural cutoff in the data to assign high or low expression status for cases (for most biomarkers that show a near normal distribution, defining optimal cutoff values to assign low or high expression status is more difficult). In a second independent cohort of patients, who also received no systemic adjuvant therapy (*n*=185), they confirmed that cancers with higher PRAME expression had poorer survival. When PRAME expression was correlated with survival in a third cohort of patients who received adjuvant chemotherapy (*n*=110), no association with outcome was seen. This suggests that high PRAME expression is predictive of poor prognosis in the absence of adjuvant chemotherapy, but is also predictive of greater sensitivity to chemotherapy (ie poor prognosis is no longer observed if patients receive adjuvant chemotherapy). Markers with similar characteristics have been reported previously including proliferative activity, histologic grade or the Oncotype DX recurrence score.

Perhaps the most important feature of this article is that these provocative observations were made entirely through *in silico* analysis of publicly available gene expression data (that was initially generated by these investigators), without any additional experiments. In the past few years, a remarkable transformation has begun to evolve in the biomarker field. Comprehensive high-throughput genomic analytical tools including mRNA gene expression profiling are increasingly applied to human cancers in an attempt to discover predictors of clinical outcome. When the main results from these studies are published, it is required by most journals to make the genomic data public. An important central repository for these data sets is the Gene Expression Omnibus (http://www.ncbi.nlm.nih.gov/geo). Among many others, there are now publicly available comprehensive gene expression data sets from cohorts of breast cancers patients who did not receive any systemic adjuvant therapy, as well as from oestrogen receptor-positive patients who were treated with adjuvant endocrine therapy only, and from patients who received preoperative chemotherapy.

These databases provide an unprecedented opportunity to rapidly evaluate almost any mRNA expression-based marker separately for prognostic, endocrine and chemotherapy response predictive values *in silico* (Andre *et al*, 2007). Studies that used to require many years to complete for immunohistochemistry-based markers can now be completed in a few weeks by a well trained investigator in basic bioinformatics methods. Not only can single genetic markers be assessed, but also any number of combinations of genes that might represent cellular regulatory pathways or other molecular functions can be tested. The multiple data sets also provide an opportunity for marker optimisation in one data set and independent validation in others.

One important caveat to this approach is that results generated by *in silico* analysis of publicly available genomic data are only as reliable as the source data itself. It is often difficult, or impossible, to validate the accuracy of clinical annotations. Technical noise due to variable quality of measurements can be substantial and systematic differences in the analytical methods including array platforms and data normalisation can make comparisons across data sets challenging. For these reasons, it is unlikely that any biomarker discovered solely through *in silico* analysis would gain wide clinical acceptance in the foreseeable future. This is a preliminary approach to the value of the markers and future studies should follow the reporting recommendations for tumour marker prognostic studies (REMARK, McShane *et al*, 2005) and standardised end points and events in clinical trials (STEEP, Hudis *et al*, 2007). It is also important to consider that if the intended clinical assay is different from the high throughput platform, validation of the new assay (eg, PCR or immunochemistry) *vs* the original microarray findings is necessary and inter- and intra-assay variation should also be provided. Nevertheless, genomic data sets and the rapidly expending bioinformatic tools to analyse them provide a unique opportunity to quickly evaluate biomarker concepts with minimal cost and identify promising markers for prospective validation on precious, nonrenewable clinical specimens.
